# Adherence to antiretroviral therapy in people living with HIV with moderate or severe mental disorder

**DOI:** 10.1038/s41598-023-30451-z

**Published:** 2023-03-02

**Authors:** Priscilla Arashiro, Camila Guadeluppe Maciel, Fernanda Paes Reis Freitas, Gabriel Serrano Ramires Koch, João Cesar Pereira da Cunha, Anderson Ravy Stolf, Anamaria Mello Miranda Paniago, Márcio José de Medeiros, Cláudia Du Bocage Santos-Pinto, Everton Falcão de Oliveira

**Affiliations:** 1 Programa de Pós-Graduação em Doenças Infecciosas e Parasitárias, Faculdade de Medicina, Universidade Federal de Mato Grosso do Sul, Campo Grande, MS Brazil; 2 Instituto Integrado de Saúde, Universidade Federal de Mato Grosso do Sul, Campo Grande, MS Brazil; 3 Hospital Universitário Maria Aparecida Pedrossiam, Universidade Federal de Mato Grosso do Sul, Campo Grande, MS Brazil; 4 Faculdade de Medicina, Universidade Federal de Mato Grosso do Sul, Campo Grande, MS Brazil; 5 Instituto Politécnico, Universidade Federal do Rio de Janeiro, Macaé, RJ Brasil

**Keywords:** Infectious diseases, Psychiatric disorders, Epidemiology

## Abstract

Human immunodeficiency virus (HIV) infection remains a serious public health concern, with an estimated 38 million people living with HIV (PLHIV). PLHIV are often affected by mental disorders at higher rate than the general population. One challenge in the control and prevention of new HIV infections is adherence to antiretroviral therapy (ART), with PLHIV with mental disorders having seemingly lower adherence than PLHIV without mental disorders. This cross-sectional study assessed adherence to ART in PLHIV with mental disorders who attended the Psychosocial Care Network health facilities in Campo Grande, Mato Grosso do Sul, Brazil, from January 2014 to December 2018. Data from health and medical databases were used to describe clinical–epidemiological profiles and adherence to ART. To assess the associated factors (potential risk or predisposing factors) with ART adherence, we used logistic regression model. Adherence was extremely low (16.4%). Factors associated with poor adherence were lack of clinical follow-up, particularly in middle-aged PLHIV. Other apparently associated factors were living on the streets and having suicidal ideation. Our findings reinforce the need for improvements in the care for PLHIV with mental disorders, especially in the integration between specialized mental health and infectious disease health facilities.

## Introduction

The human immunodeficiency virus (HIV) infection and acquired immunodeficiency syndrome (AIDS) epidemics represent a multifaceted phenomenon whose regional sub-epidemic occurrence in different regions of the world depends, among others, on individual and collective human behaviors^1^. According to the Joint United Nations Programme on HIV/AIDS (UNAIDS) report^2^, 38.4 million individuals globally were living with HIV in 2021, with 1.5 million newly infected individuals in this same year. In Brazil, 1,045,355 cases of HIV/AIDS have been reported between 1980 and June 2021. Between 2017 and 2021, an annual average of 36,800 new cases of HIV/AIDS has been reported in Brazil^3^. Regarding mental and psychiatric disorders, the occurrence of these disorders is 1.5–8 times higher in people living with HIV (PLHIV) than in the general or uninfected population^4^.

The association between HIV/AIDS and mental disorders is complex and multifactorial^4^; some psychiatric outcomes can be associated with the neurotropism of the virus and psychosocial factors. Among these neuropsychiatric outcomes, the direct effects of HIV on the central nervous system and consequences of opportunistic diseases, tumors, and cerebrovascular diseases, and complications resulting from antiretroviral therapy (ART) have been cited^5^. The psychosocial consequences related to HIV infection highlight the significant emotional suffering experienced by PLHIV due to several issues, such as discrimination and fear of illness, both of which directly interfere with the health and quality of life of PLHIV^6^. Inverse causality should also be considered when assessing the association between HIV and mental disorders, since, owing to decreased self-care and lack of social support, individuals with severe mental disorders are at a greater risk of HIV infection than those without mental disorders^7^.

Within this context, the treatment of moderate and severe mental disorders and HIV/AIDS is complex due to the clinical and behavioral characteristics of individuals with these conditions^8^. The burden of both diagnoses^9^ and the prejudice and stigma faced by individuals with these diagnoses characterize them as neglected populations^10^.

In Brazil, individuals with mental disorders can be assisted by the Psychosocial Care Network (*Rede de Atenção Psicossocial*, RAPS in the Brazilian abbreviation) of the National Unified Health System (*Sistema Único de Saúde*, SUS in the Brazilian abbreviation). The RAPS is a thematic component of SUS implemented in 2011 and provides a wide range of services at all levels of health care, from primary care to tertiary hospital level. One of the highlights of this mental health care network is the Psychosocial Care Centers (*Centro de Atenção Psicossocial*, CAPS in the Brazilian abbreviation), which provide open and community health services to those with moderate and severe mental disorders, including those with needs arising from alcohol and drug abuse, whether in crisis situations or in psychosocial rehabilitation processes, thereby promoting comprehensive care for different demands^11,12^. CAPS are classified according to the type of service provided and to the population size of the municipality where they are located: types I, II and III meet different demands of mental disorders and differ by the population size of the municipality, with type I found in small cities and type III in large cities. The CAPS AD and CAPSi types address mental disorders related to alcohol and drug use and children, respectively. Another component of the RAPS is the Street Office teams (eCR) that provide care to the homeless population^11^. These two components of RAPS (CAPS and eCR) are of great importance for approaching individuals with mental disorders, including those living with HIV/AIDS.

The UNAIDS has emphasized the need for better integration between mental health and HIV health facilities, highlighting that scarcely any health services address the HIV-related needs of individuals living with mental health problems nor the mental health problems of PLHIV^13^.

HIV/AIDS treatment consists of the continuous and regular use of ART, which is essential for increasing survival^14–17^. Good adherence to ART includes taking the relevant medication correctly, following the dosage for a pre-established amount of time, and having a periodic attendance at health services^18^. The first-choice regimens available free at SUS in Brazil can maintain viral suppression. Although there is no consensus on the adherence rates necessary to reach the viral suppression, many studies consider a good adherence to be when patients use between 80 and 100% of the prescribed doses^19^. Although adherence has been widely studied^20^, data on medication adherence in HIV-infected patients with psychiatric comorbidities remain limited^21–23^. Considering this context, the aim of the present study is to evaluate the ART adherence and its associated factors in PLHIV with moderate or severe mental disorders who were assisted by the Psychosocial Care Network (RAPS) health facilities of Campo Grande, Mato Grosso do Sul (MS), Brazil. We also evaluated factors associated with two laboratory markers of treatment response, the HIV load and the CD4 lymphocyte count.

## Methods

### Study design, setting, and data source

This cross-sectional study used data of PLHIV assisted by the RAPS in Campo Grande, MS, from January 2014 to December 2018.

Campo Grande has an estimated population of 916,001 inhabitants (https://cidades.ibge.gov.br/brasil/ms/campo-grande/panorama) and has a structured RAPS, including five CAPS intended for adult care, which are as follows: one CAPS alcohol and drugs (CAPS AD IV) and four CAPS III; an eCR, linked to primary care. In addition, it has a Specialized Center for the Treatment of Infectious and Parasitic Diseases and the Infectious and Parasitic Diseases Unit of the University Hospital of the Federal University of Mato Grosso do Sul, Brazil. Since 2016, rapid HIV testing initiatives have begun in all health units, including primary health care, prior to which HIV testing was performed only in centers specializing in infectious diseases. In 2018, the service was expanded to mental health units, while rapid tests have been conducted on the population under their care.

Individuals aged ≥ 18 years who were residents of Campo Grande, diagnosed with HIV/AIDS, and could be linked to at least one of the RAPS points (CAPS or eCR) were included in this study. Individuals with medical records and/or registration/notification forms with incomplete data were excluded.

The first step to identify and locate the population eligible for the study was to combine, using the manual record linkage, two databases from different health information systems in Brazil: National System of Disease Notification—SINAN (database for notifiable diseases; period used: 1984, when AIDS reporting began in Brazil, to 2018) and Outpatient Information System—SIA-SUS (database for outpatient and specialized care, including care provided at CAPS; period used: 2014 to 2018). Names, national health card numbers, date of birth, and mother’s name used for the manual record linkage to identify individuals appearing in both databases were used.

In the second stage, data on individuals’ clinical–epidemiological profiles were collected from the HIV/AIDS notification forms in SINAN, medical records held at the RAPS care points, and centers specializing in infectious and parasitic diseases. The electronic medical records available in the Information Management System of the Municipal Health Department and the eCR service reports were also reviewed.

Data regarding the ART dispensing history and the number individuals living with HIV who were on antiretroviral treatment during the study period were extracted from the Brazilian Medication Logistics Control System—SICLOM. HIV viral load and CD4+ cell counts were obtained from the Laboratory Test Control System of the National Counting Network for CD4+/CD8+ Lymphocytes and HIV Viral Load—SISCEL.

### Outcome measure and definitions

Adherence to ART, the main outcome of this study, was assessed by the HIV medicines refill data during 2014 to 2018. We defined adherence as monthly and regular ART refills with a maximum delay of 6 days; hence delays that are ≥ 7 days were defined as non-adherence. Refills occurring before the expected date of refill (i.e., before ending the stock of medicines in the patient’s possession) were carried forward to subsequent periods^24^, except for switched therapy during the follow-up.

Considering that virological response and immunological status may be a consequence of treatment adherence or signal of advanced disease, not adherence itself, they were assessed separately. The latest measurements available in SISCEL were used to assess viral (undetectable viral load) and immunological (CD4 > 200 cells/mm^3^) response at the end of the follow-up period.

Mental disorders were categorized in two ways based on the tenth edition of the International Classification of Diseases. The first categorization included the following distribution:

- F00–F09: organic mental disorders
- F10–F19: mental and behavioral disorders due to psychoactive substance use
- F20–F29: schizophrenia, schizotypal disorders, and delusional disorders
- F30–F39: mood disorders (affective)
- F40–F48: neurotic disorders, stress-related disorders, and somatoform disorders
- F60–F69: behavioral syndromes associated with physiological dysfunctions and physical factors
- F70–F79: adult personality and behavior disorders

The second categorization grouped mental disorders into four categories: anxiety (F40–F48), mood (F30–F39), substance use (F10–F19), and other disorders, which grouped disorders not included in the first categorization.

Other clinical–epidemiological data assessed were time of HIV infection (years), possible mode of transmission of HIV and sexual behavior/practice, co-infections, having regular follow-up at CAPS and services specialized in infectious and parasitic diseases (at least one appointment with an infectious disease doctor every 6 months), follow-up regimen at RAPS (shelter, outpatient, shelter and outpatient), drug use, alcohol use, and suicidal ideation.

Sociodemographic data included were sex, age, race/skin color, educational level, and history of homelessness.

### Data analysis

To characterize the study population, the qualitative variables were described using the measures number of occurrences (*n*) and proportions (%), the data were presented in frequency distributions. For continuous variables, the descriptive measures were the minimum, maximum, first quartile, third quartile, mean, median, and standard deviation.

The *t* test for paired data was used to assess the differences of means of viral load and CD4 cells between the beginning and end of period of follow up.

An univariable analysis was used to analyze the association between each independent variable and adherence to ART, plasma HIV load and CD4 count. The Mann–Whitney *U* test was used in the case of continuous variables and Fisher's exact test for categorical variables. The significance level adopted for all hypothesis tests was 5% (α = 0.05). Additionally, the odds ratio was calculated, including the respective 95% confidence interval.

A multivariate logistic regression model was used to investigate the variables related with adherence to ART. The stepwise algorithm was used for model selection using the Akaike Information Criterion (AIC) in both directions. As a measure of goodness of fit, the Hosmer and Lemeshow test was adopted.

The analysis was performed using R software version 4.0.4 (https://www.r-project.org/), and the following packages were used: *tidyverse*, *descr*, and *generalhoslem*.

### Ethics declarations

This study was approved by the Human Subject Research Ethics Committee of the Federal University of Mato Grosso do Sul, Brazil (protocol number: 3416555; CAAE: 15431819.3.0000.0021). All procedures were performed in accordance with relevant guidelines and regulations. Informed consent was obtained from all participants.

## Results

### Sociodemographic characteristics

A total of 8642 cases of HIV/AIDS were reported to the SINAN from 1984 to 2018 in Campo Grande. In the SIA-SUS, from 2014 to 2018, 10,699 outpatient consultations were located at all RAPS health facilities. Cross-referencing these systems resulted in 80 occurrences of patients present in both databases. Of these, four were excluded from this study: one for being under 18 years old; two for having incomplete medical records that made it impossible to collect the data necessary for the study; one who, despite having a history of attendance at the RAPS facilities, did not have a diagnosis of a moderate or severe mental disorder. Therefore, 76 participants were included in this study.

During the study period, 1972 PLHIV were on antiretroviral treatment. Considering this number as an approximation of the number of PLHIV in the setting and study period, the prevalence of moderate or severe mental disorders was 38.5 cases per 1000 PLHIV. Regarding sex, 31.6% (*n* = 24) and 68.4% (*n* = 52) were males and females, respectively. The mean of age at the time of HIV/AIDS diagnosis was 35.6 years (median = 34 years; standard deviation = 10.8), and at the end of the study was 39.9 years (median = 39 years; standard deviation = 11.8). Browns (*n* = 30, 39.5%) and Whites (*n* = 28, 36.8%) represented the most frequent categories. Other data are presented in Table 1.

**Table 1:** Sociodemographic data of the study population (*n* = 76).

Variable	n	%
**Sex**		
Female	24	31.6
Male	52	68.4
**Age (year) on December 31, 2018**		
18–25	9	11.8
26–40	30	39.5
41–60	35	46.1
61 and more	2	2.6
**Age (year) at the time of the HIV/AIDS diagnosis report**		
18–25	14	18.4
26–40	37	48.7
41–60	24	31.6
61 and more	1	1.3
**Race or skin color**		
Brown	30	39.5
White	28	36.8
Black	7	9.2
Yellow	7	9.2
Ignored	4	5.3
**Educational level**		
Illiterate	3	3.9
Elementary school (1–9 years of study)	23	30.3
High school (10–12 years of study)	8	10.5
Higher education (over 13 years of study)	10	13.2
Ignored	32	42.1
**Homeless**		
Yes	17	22.4
No	59	77.6

Data source: Notifiable Diseases Information System (SINAN).

### Clinical–epidemiological characteristics

The clinical–epidemiological data are listed in Table 2. The probable mode of HIV infection was non-vertical (sexual or parenteral transmission) in 90.7% (*n* = 69) of cases, while the sexual behaviors were homosexual (*n* = 10, 13.1%), heterosexual (*n* = 55, 72.3%), and bisexual practices (*n* = 5, 6.5%).

**Table 2:** Clinical–epidemiological data of the study population (*n* = 76).

Variable	n	%
**Time of HIV infection (years)**		
Less than 1	17	22.3
1–5	36	47.3
5–10	13	17.1
10–20	7	9.2
21 and more	3	3.9
**Possible mode of transmission of HIV**		
Vertical	0	0
Non-vertical	69	90.7
Ignored	7	9.2
**Sexual behavior/practice**		
Men who have sex with women	32	42.1
Men who have sex with men	10	13.1
Men who have sex with women and men	5	6.5
Women who have sex with men	23	30.2
Ignored	6	7.8
**Injection drug use**		
Yes	9	11.8
No	43	56.5
Ignored	24	31.5
Co-infections*		
Tuberculosis	9	18.3
Bacterial lesions located in the dermis	7	14.2
Gastroenteritis	6	12.2
Pneumonia	5	10.0
Herpes zoster	4	8.1
Syphilis	4	8.1
Others**	14	28.5
**ART regimens**		
TDF + 3TC + DTG	21	29.5
TDF + 3TC + EFV	21	29.5
ATV + TDF + 3 TC + RTV	8	11.2
AZT + 3TC + ATV + RTV	6	8.4
AZT + 3TC + EFV	4	5.6
AZT + 3TC + LPV/r	3	4.2
RAL + ETR + RTV + DRV	1	1.4
AZT + 3TC + TDF + LPV/r	1	1.4
3TC + TDF + LPV/r	1	1.4
NVP + TDF + 3TC	1	1.4
FPV + TDF + 3TC + RTV	1	1.4
RAL + ETR + TDF + 3TC + TRV + DRV	1	1.4
RAL + TDF + 3TC	1	1.4
RTV + DRV + DTG + ETR	1	1.4

Data source: Notifiable Diseases Information System (SINAN) and outpatient and hospital records.

*Data from medical records.

**Candidiasis, warty lesions and/or genital warts, neurotoxoplasmosis, neurocryptococcosis, vulvovaginitis, histoplasmosis, gonorrhea, and myiasis.

*TDF* tenofovir, *3TC* lamivudine, *EFV* efavirenz, *DTG* dolutegravir, *ATV* atazanavir, *RTV* ritonavir, *LPV/r* ritonavir-boosted lopinavir, *AZT* zidovudine, *NVP* nevirapine, *RAL* raltegravir, *ETR* etravirine, *DRV* darunavir, *FPV* fosamprenavir.

Regarding injection drug use, 11.8% (*n* = 9) claimed to be users. This field was not filled in 31.5% (*n* = 24) of the notification forms in SINAN. We observed a higher frequency of reports of drug use at 32 based on the review of medical records, including inhaled and injected drugs. Base paste was the most prevalent drug (*n* = 25), followed by crack (*n* = 5) and cocaine (*n* = 2). Marijuana was described as an associated drug rather than as the main drug in 12 cases.

Based on the review of medical records, 29 patients had one record of HIV/AIDS-associated infection (co-infection) during the study period, 10 had two co-infections, and 3 had ≥ three co-infections.

Among the 76 participants included in the study, 4 were not registered with SICLOM and therefore could not be located, 3 were registered but never took medication, and 8 were registered but did not take antiretroviral medication during the analysis period of this study. Therefore, the data presented in this section are of 61 PLHIV.

The dispensed antiretroviral medicines included tenofovir (TDF), lamivudine (3TC), efavirenz (EFV), dolutegravir (DTG), atazanavir, ritonavir (RTV), RTV-boosted lopinavir, zidovudine, nevirapine, raltegravir, etravirine, darunavir, and fosamprenavir. The predominant regimens, accounting for 59% of the dispensations, were TDF + 3TC + DTG and TDF + 3TC + EFV. During the follow-up, regimen switching was observed in 33 individuals. The most common cause for switching was adverse events to ART (*n* = 7), followed by virological failure (*n* = 2) and co-infection with tuberculosis (*n* = 1). The other causes of switching were not available at SICLOM.

Frequencies and descriptive measures of HIV viral loads and CD4+ cells for the first and last available reports during the follow-up are summarized in Table 3. There was an increase in participants with an undetectable viral load at the end of the study period, but no mean difference of HIV loads at the beginning and end of study period (paired *t* test = 0.44; p-value = 0.66). The number of participants with immunological failure at the end decreased and with differences between the means of CD4 cells at the beginning and end of the follow-up (paired *t* test = − 4.92; p-value < 0.001).

**Table 3:** Frequencies and descriptive measures of HIV viral load and CD4+ cells according to initial and final study periods (*n* = 76).

	Baseline^A^		Endpoint^B^	
	*n*	%	*n*	%
**Viral load**				
Not detected or less than 40	18	26.9	44	65.7
Detectable	49	73.1	23	34.3
No data during the study period*	9	–	9	–

	copies/mL		copies/mL	
Minimum	48	–	48	–
Maximum	678,308	–	1,298,458	–
Mean	103,263	–	97,278.48	–
Median	22,865	–	6195	–
Standard deviation	160,756.5	–	274,774.3	–

	*n*	%	*n*	%
**CD4+ cells**				
Less than 200	16	23.5	11	16.2
201–350	9	13.2	10	14.7
350–500	13	19.1	10	14.7
Higher than 500	30	44.1	37	54.4
No data during the study period*	8	–	8	–

	cells/mm^3^		cells/mm^3^	
Minimum	9	–	8	
Maximum	1092	–	1620	–
Mean	447.53	–	586.1	–
Median	417	–	510	–
Standard deviation	273.5	–	381.5	–

Data source: Laboratory Test Control System of the National Network for CD4+ Lymphocyte Count and HIV Viral Load (SISCEL).

*Not included in descriptive analysis.

^A^Refers to the closest measure of 2014.

^B^Refers to the closest measure of 2018.

### ART and factors associated with adherence

Most PLHIV were classified as non-adherent (*n* = 51, 83.6%). In the univariate analysis (Table 4), adherence was significantly associated with age and mental disorders, with emphasis on those related to substance use. Sex, race/skin color, having regular follow-up at services specialized in infectious and parasitic diseases, and mental disorder according to ICD-10 were also included in the multivariate analysis.

**Table 4:** Adherence to ART according to clinical–epidemiological data (*n* = 61).

	Adherence		*p*-value*	OR (95% CI)	
	No (%)	Yes (%)		Univariable	Multivariable
**Age**					
Mean (SD)	33.0 (14.4)	42.4 (11.3)	0.021	1.08 (1.01–1.16)	1.13 (1.05–1.26)
Median (IQR)	29.5 (22.5–37.8)	43.0 (36.0–50.0)		–	–
**Sex**					
Female	1 (5.3)	18 (94.7)	0.151	Reference	–
Male	9 (21.4)	33 (78.6)		0.20 (0.01–1.21)	–
**Race or skin color**					
White	5 (22.7)	17 (77.3)	0.175	Reference	–
Black	1 (20.0)	4 (80.0)		1.18 (0.13–25.85)	–
Yellow	2 (40.0)	3 (60.0)		0.44 (0.06–4.05)	–
Brown	2 (7.7)	24 (92.3)		3.53 (0.67–26.73)	–
**Educational level**					
Elementary school	4 (22.2)	14 (77.8)	0.598	–	–
High school	0 (0.0)	5 (100.0)		–	–
Higher education	1 (10.0)	9 (90.0)		–	–
Illiterate	0 (0.0)	1 (100.0)		–	–
**Regular follow-up at CAPS**					
No	5 (13.5)	32 (86.5)	0.495	Reference	–
Yes	5 (20.8)	19 (79.2)		0.59 (0.15–2.39)	–
**Homeless**					
No	9 (18.8)	39 (81.2)	0.674	Reference	–
Yes	1 (7.7)	12 (92.3)		2.77 (0.45–53.71)	–
**Abuse of alcohol**					
No	7 (22.6)	24 (77.4)	0.301	Reference	–
Yes	3 (10.0)	27 (90.0)		2.62 (0.65–13.25)	–
**Use of drugs**					
No	6 (17.1)	29 (82.9)	1.000	Reference	
Yes	4 (15.4)	22 (84.6)		1.14 (0.29–4.91)	
**Regular follow-up in the specialized outpatient infectious disease facility**					
No	2 (6.5)	29 (93.5)	0.071	Reference	Reference
Yes	7 (25.0)	21 (75.0)		0.21 (0.03–0.96)	0.14 (0.02–0.76)
**Suicidal ideation**					
No	5 (11.9)	37 (88.1)	0.261	Reference	–
Yes	5 (26.3)	14 (73.7)		0.38 (0.09–1.55)	–
**Service regimen at RAPS**					
Shelter	0 (0.0)	5 (100.0)	0.672	–	–
Outpatient	4 (14.8)	23 (85.2)		–	–
Shelter and outpatient	6 (20.7)	23 (79.3)		–	–
**Mental disorder**					
Anxiety	4 (50.0)	4 (50.0)	0.034	Reference	–
Humor	2 (20.0)	8 (80.0)		4.00 (0.54–39.54)	–
Related to substance use	4 (11.8)	30 (88.2)		7.50 (1.33–46.42)	–
Others	0 (0.0)	9 (100.0)		–	–
**ICD-10 categories for mental disorders**					
F00–F09	0 (0.0)	3 (100.0)	0.144	–	–
F10–F19	4 (11.8)	30 (88.2)		–	–
F20–F29	0 (0.0)	5 (100.0)		–	–
F30–F39	2 (20.0)	8 (80.0)		–	–
F40–F48	4 (50.0)	4 (50.0)		–	–
F60–F69	0 (0.0)	1 (100.0)		–	–
**Co-infection**					
No	6 (16.7)	30 (83.3)	1.000	Reference	–
Yes	4 (16.0)	21 (84.0)		1.05 (0.27–4.54)	–

Data source: Medication Logistics Control System (SICLOM) and data obtained from the review of outpatient and hospital records of follow-up services in 2021.

*Fisher’s exact test.

*OR* odds ratio, *95% CI* 95% confidence interval, *SD* standard deviation, *IQR* interquantile range, *CAPS* Psychosocial Care Centers, *RAPS* Psychosocial Care Network, *ICD-10* 10th edition of the International Classification of Diseases.

To assess the effects of covariates on the outcome while considering possible confounding and interactions, the logistic regression model was adopted. As shown in Table 4, only age, regular follow-up at services specializing in infectious and parasitic diseases, and mental disorders showed a significance of < 10% for the univariable models (univariable OR). However, in the multivariable model (multivariable OR), according to variable selection via the stepwise algorithm, only the main effects of age and regular follow-up at services specializing in infectious and parasitic diseases remained statistically significant. The interaction between age and regular follow-up at services specializing in infectious and parasitic diseases was not significant (Table 5).

**Table 5:** Deviance analysis for ART adherence data.

Model	Residuals deviance	Residuals degrees of freedom	Deviance	Degrees of freedom	*p*-value
Constant	50.397	58	–	–	–
Regular follow-up at services specialized in infectious and parasitic diseases	46.322	57	4.075	1	0.046
Age	34.733	56	11.589	1	0.001
Regular follow-up at services specialized in infectious and parasitic diseases + Age + Interaction	33.038	55	1.695	1	0.193

Table 6 presents estimated results of the logistic regression; the association between adherence and covariates of age and regular follow-up at services specializing in infectious and parasitic diseases was modeled, emphasizing the potential effect of age as a risk factor (β_*i*_ = 0.13; OR = 1.14; 95% CI 1.05–1.26) and the protective effect of regular follow-up for adherence (β_*i*_ = − 1.98; OR = 0.14; 95% CI 0.02–0.76). According to the goodness of fit by Hosmer and Lemeshow, the *p*-value (0.639) was not significant, indicating that the model was well adjusted.

**Table 6:** Regression results for adherence to ART (outcome = adherence (no)).

Covariates	β Coefficient (standard error)
Constant	− 1.626 (1.550)
Regular follow-up in the specialized outpatient infectious disease facility (yes)	− 1.978* (0.944)
Age	0.126** (0.044)

Log Likelihood: − 17.367; Akaike Information Criterion: 40.733.

*p-value < 0.05; **p-value < 0.001.

### Factors associated with HIV viral loads and CD4+ cells at the end period

The regular follow-up in specialized services was associated with undetectable viral load, suggesting that frequent access to specialized services results in better viremia control (Supplementary Table S1). Being homeless seemed to be associated with detectable viral load, but the difference was only significant by one-tailed Fisher's exact test (*p*-value = 0.037), and not significant by two-tailed Fisher’s exact test (*p*-value = 0.067). Similarly, having suicidal ideation seemed to be associated with detectable viral load, but the difference was significant by one-tailed Fischer’s exact test (*p*-value = 0.061), and not by the two tailed test (*p*-value = 0.045). In addition, the OR demonstrated that being homeless is a potential risk factor for viral failure (OR = 3.4; 95% CI 1.1–11.3).

Although viral load may be a consequence of adherence, and the design of this study does not allow for the assessment of cause-effect relationships, our data demonstrated an association between viral load and adherence to ART (*p*-value = 0.03).

The regular follow-up in specialized services was associated with higher CD4 cell counts. Likewise, people with coinfections presented lower CD4 counts (Supplementary Table S2).

### Outpatient follow-up and adherence to ART

A total of 70 records of PLHIV were found in health facilities specializing in infectious diseases. Among these, the records of regular follow-ups in the infectious disease facility were observed in 30 (42.9%) participants, whereas 40 (57.1%) participants had irregular follow-ups. When analyzing the cases of irregular follow-ups, 34 had records of delays in ART refill, of which 27 were also reported as abandoning ART during the study period (more than 100 days to refill). That is, all cases of ART abandonment had irregular follow-ups since such cases had no records of specialized consultations every 6 months.

Among the 17 PLHIV living on the streets, only one was followed at the specialized infectious disease service, 16 presented delayed pharmacy ART refills, 12 had already abandoned treatment. In the one case of regular ART refill, the refill was made by the eCR.

## Discussion

Antiretroviral therapy has revolutionized HIV treatment, but its success is dependent on adherence. Studies utilizing medication dispensation data from high and middle-income countries have reported ART adherence ranging from 61.8 to 83.6%^24–26^. Here, we report much lower ART adherence rates (16.4%) for a historically neglected and underreported population: those burdened simultaneously with HIV/AIDS and psychiatric conditions^9,10,27^. Few studies have explored adherence in similar populations, but we suspect that this double burden of high proportion of non-adherence to ART in PLHIV with moderate or severe mental disorders to be a common phenomenon. Due to the complexity and variety of clinical outcomes in mental disorders, analytical studies with appropriate control groups are difficult to design and perform to estimate the risk of non-adherence to ART according to psychiatric diagnosis.

Psychiatric disorders account for 18% of ART^28^ and several studies have demonstrated the association between mental disorders and discontinuation of ART^23,28–30^. For example, depressive symptoms were strongly associated with poor adherence to treatment and uncontrolled viral replication^6,31^. The interplay between various psychiatric conditions and HIV infection is complex. Mental disorders may make initial infection more likely, and infection control with effective ART therapy more challenging^6^.

We discuss some hypotheses that may help explain our main finding: that ART adherence is lower in those with moderate and severe mental disorders compared to the entire population living with HIV/AIDS. First, as we observed that lack of regular medical follow-up in the service specialized in infectious diseases was associated with low adherence to ART, we suggest that the demand for infectious disease physicians might exceed their capacity^32^. Indeed, data from the National Register of Health Establishments of Brazil shows that infectious diseases physicians/PLHIV ratio during the study period in Campo Grande was 0.006 (mean number of physicians = 11.9; PLHIV with record of dispensation = 1972)^32^. In the same time there was also a reduction in the number of specialist physicians and their workloads^32^. Further contributing to this capacity issue is that specialized services are busy with other duties, as they provide care for other infectious diseases in outpatient and inpatient settings, while also being responsible for teaching and research activities for multiprofessional residency programs and coordination of the hospital infection control commission. We suggest that these issues impacted the quick and timely access to follow-up while potentially contributing to the discontinuation of treatment, despite protocols from the Ministry of Health that include searching for patients when treatment are abandoned. Specialized outpatient care is an operational bottleneck for SUS operations in Brazil^33,34^, which is characterized by demand exceeding capacity and prolonged wait times before consultations, including for time-sensitive issues relating to mental health or infectious diseases.

The low number of infectious diseases physicians observed in our study may have directly impacted the patient follow-up that was recommended by the Brazilian Ministry of Health^35^, consequently affecting their adherence to ART. If generalizable by studies in other geographies^20,30^, our results demonstrate the importance of access to follow-up care for good adherence to ART, especially in patients with mental health comorbidities.

A second potential cause underlying our observation is that various specialized services (e.g. infectious diseases facilities, RAPS point of care) are not integrated. Medical records are not shared between different services and specialties, and there is little coordination between these units in the active search for patients with delays in their medication dispensing. Highlighting the lack of interaction between infectious disease services and RAPS is that patients common to both services had to be identified manually. An exception to this was found in patients sheltered in mental health services facilities (or accompanied with the office team on the street), where we found examples of referral and counter-referral between different services. But generally, this trend of information fragmentation and the disintegration of systems and care units is common throughout Brazil. This is especially in the case with the Department of HIV/AIDS, which had not made advances to the national information system or in the integration of care^36^. Cooperation between services positively influence treatment adherence throughout the treatment course: from the first visit with an infectious disease physician, through scheduling and conducting various appointments. Data suggest that the organization of health services and effective communication between different specialties (infectious diseases and psychiatry) are important for ART adherence.

Third, we suggest that the centralization of ART dispensing to only reference centers of infectious diseases has generated accessibility barriers to follow-up by specialized services and impacting continuation of ART therapy^34,37^. The decentralization of HIV/AIDS services to primary care has been successfully introduced into many community-based programs since the 1990s in high^38^ and low-income settings^39,40^, strengthening primary care and improving ART adherence.

The prevalence of moderate or severe mental disorders among PLHIV estimated in our study is within ranges reported in studies conducted in several countries (0.93–57%)^10,41–48^. Notably, our data only consider mental health care provided in the SUS, and the actual prevalence of moderate and severe mental disorders in PLHIV in the study area could be higher than reported. The prevalence of moderate and severe mental disorders in the general population of a large Brazilian city is estimated at around 3.3 per 1000 people city^49^, while hospital admissions for mental disorders in Brazil was 77.44 per 100,000 between 2008 and 2018^50^. Certainly, our low observed prevalence could reflect low access to mental health services for individuals living with HIV and the low capacity of the mental health care network to provide care to those people. Other authors have showed that mental health follow-up was important for the continuation of ART in PLHIV^51^.

Patient age also had a significant impact on ART adherence and our findings showed that age is associated with lower ART adherence. These results are similar to those reported by Valle Camelo et al.^52^, who found that individuals over 40 years old were more likely to have inaccurate perceptions and conceptions about the burden of AIDS and the importance of its treatment for control (i.e. adherence to ART) compared with younger individuals, reinforcing the importance of health education especially for older adults and their families/caregivers, in addition to training health professionals, to better meet the demands of aging populations.

The epidemiological profile of the study participants showed a similar pattern of PLHIV in Brazil, with a higher prevalence of HIV/AIDS among males of white and brown color^3^. The differences found refer to age and sexual orientation or behavior from national data on PLHIV, which indicate that the highest frequency of PLHIV is observed in men who have sex with men^3^. One explanation for this difference is the influence of drug use on HIV transmission among the study participants, because of syringe sharing. In this context, variables related to sexual practice may be less relevant. However, the number of patients described must also be considered since it may result in the study sample not being representative of the whole population.

The literature provides data on the high prevalence of homelessness among PLHIV^53^. It is common for individuals living with diseases (e.g. AIDS, leprosy, mental disorders, physical disabilities) and their associated social stigmas to have families having limited resources to meet the financial costs associated with these conditions. This in part contributes to these individuals being cared for by public institutions or living on the streets^54^. Homelessness is a risk for many health conditions, especially those caused by infectious diseases and mental disorders^55^.

This study had limitations, including reliance on incomplete medical records necessitating manual analyses. Electronic medical records were officially adopted in 2013, but have not yet been implemented in most Brazilian municipalities^36^. Furthermore, measures of adherence based on dispensing and self-report are not a guarantee of ART usage^19^; thus, other measures, such as clinical follow-up examinations, should be considered in future studies. Additionally, this study only included patients assisted in the SUS; hence, even though this study focuses on a population that is often ignored, it may not represent all PLHIV and moderate to severe mental illness. However, due to the history of coverage of the HIV/AIDS program in Brazil, most PLHIV are followed up by the public health system^35,56^, what can reduce this particular weakness of the study. Finally, as this is a descriptive study with a small population and sample size, the association analyzes presented do not allow causal inferences nor are they a robust indication of risk and/or protective factors. However, the questions generated from the results of this study should be explored in future analytical studies with this population.

Concerningly, some recent changes to the public policy in Brazil risk further worsening of healthcare services. Dramatic health policy changes made from 2019 to 2022 have made providing comprehensive care difficult, especially for neglected populations. In addition, the HIV/AIDS Department was abolished in 2019, which negatively affected the financing and services, causing a major setback in the care for PLHIV. In psychosocial care, approximately 15 regulations were proposed in Brazil, leading to the so-called “New National Mental Health Policy”^57,58^ between 2016 and 2019. The redefinition of a new model of care was offered by the RAPS, but is limited as it does not consider community-based primary care projects, instead focusing only on hospital-centered care, as evidenced by the drastic reduction in the pace of implementation of CAPS between 2016 and 2018, the worst period since the beginning of the psychiatric reform^58^. In addition, there is the stimulus for the creation of therapeutic communities with the expansion of financial support and the number of vacancies for patients, in a clear movement to reinstate isolation practices^57^. Finally, the COVID-19 pandemic cannot go unmentioned as it increased the incidence of mental disorders coupled with the worsening of pre-existing conditions worldwide^59^.

A great double burden faces those who live with HIV/AIDS and mental disorders, contributing to feelings of both invisibility and stigma^10,58^. Therefore, improvements in the care network for PLHIV with mental disorders remains essential to ensure adherence to ART, especially during early diagnosis and intervention. These changes could reduce the global burden of these diseases and improve the quality of life of affected individuals.

## Conclusion

Our study, which investigated a population affected by a double burden of diseases in a socially vulnerable population that had high prevalence of behavioral disorders related to substance and alcohol abuse, co-infections, and suicidal ideation, reinforces the importance of priority action for the most vulnerable populations. We found homelessness, substance abuse, and irregular medical follow-ups (mental health and infectious disease) to be associated with poor ART adherence.

Mental illness seems to have greatly affected the adherence of people living with HIV, and there must be an improvement in the availability and quality of services aimed at this population. Without adherence to antiretroviral therapy, these patients will have higher morbidity and mortality and will overload services. Our findings can help influence the strategic actions and integrations of healthcare teams, while highlighting the importance of developing innovative approaches to solve complex problem faced by those with combined mental health and infectious disease demands. We are challenged to prioritize projects and implement policies aimed at protecting these vulnerable populations.

## Supplementary Information

Supplementary Tables.

## Data Availability

All relevant data that support the findings of this study are presented within the manuscript and its additional files.
