# Supplementary material for: Adherence to antiretroviral therapy in people living with HIV with moderate or severe mental disorder
Source: Sci Rep. 2023 Mar 2;13:3569. doi: 10.1038/s41598-023-30451-z (PMC9980869; doi:10.1038/s41598-023-30451-z)
Supplement: Supplementary file 1 — Supplementary Tables. [file 41598_2023_30451_MOESM1_ESM.pdf]

# Adherence to antiretroviral therapy in people living with HIV with moderate or severe mental disorder

Priscilla Arashiro, Camila Guadeluppe Maciel, Fernanda Paes Reis Freitas, Gabriel Serrano Ramires Koch, João César Pereira da Cunha, Anderson Ravy Stolf, Anamaria Mello Miranda Paniago, Márcio José de Medeiros, Cláudia Du Bocage Santos-Pinto, Everton Falcão de Oliveira

## Supplementary Table S1. Viral load according to clinical–epidemiological data

| | **Viral load (copies/mL)** | | ***p*–value*** | **OR (95% CI)** |
|---|---|---|---|---|
| | **Undetectable (%)** | **Higher than 40 (%)** | | |
| **Age** | | | | |
| Mean (SD) | 39.7 (12.3) | 41.6 (12.4) | 0.702 | 1.01 (0.97–1.06) |
| Meadian (IQR) | 40.0 (29.8–48.5) | 41.0 (33.0–50.0) | | |
| **Sex** | | | | |
| Female | 13 (65.0) | 7 (35.0) | 1.000 | Reference |
| Male | 31 (66.0) | 16 (34.0) | | 0.96 (0.32–2.99) |
| **Race or skin color** | | | | |
| White | 16 (66.7) | 8 (33.3) | 0.712 | Reference |
| Black | 3 (50.0) | 3 (50.0) | | 2.00 (0.31–13.13) |
| Yellow | 5 (83.3) | 1 (16.7) | | 0.40 (0.02–3.07) |
| Brown | 19 (67.9) | 9 (32.1) | | 0.95 (0.29–3.07) |
| **Educational level** | | | | |
| Elementary school | 12 (57.1) | 9 (42.9) | 0.565 | - |
| High school | 3 (60.0) | 2 (40.0) | | - |
| Higher education | 6 (60.0) | 4 (40.0) | | - |
| Illiterate | 0 (0.0) | 2 (100.0) | | - |

| | | | | |
|---|---|---|---|---|
| **Regular follow–up at CAPS** | | | | |
| No | 26 (63.4) | 15 (36.6) | 0.793 | Reference |
| Yes | 18 (69.2) | 8 (30.8) | | 0.77 (0.26–2.17) |
| **Homeless** | | | | |
| No | 37 (72.5) | 14 (27.5) | 0.067 | Reference |
| Yes | 7 (43.8) | 9 (56.2) | | 3.40 (1.07–11.28) |
| **Abuse of alcohol** | | | | |
| No | 24 (70.6) | 10 (29.4) | 0.447 | Reference |
| Yes | 20 (60.6) | 13 (39.4) | | 1.56 (0.57–4.39) |
| **Use of drugs** | | | | |
| No | 25 (67.6) | 12 (32.4) | 0.798 | Reference |
| Yes | 19 (63.3) | 11 (36.7) | | 1.21 (0.43–3.34) |
| **Regular follow–up in the specialized outpatient infectious disease facility** | | | | |
| No | 14 (40.0) | 21 (60.0) | <0.001 | - |
| Yes | 28 (100.0) | 0 (0.0) | | - |
| **Suicidal ideation** | | | | |
| No | 26 (57.8) | 19 (42.2) | 0.061 | Reference |
| Yes | 18 (81.8) | 4 (18.2) | | 0.30 (0.08–0.97) |
| **Service regimen at RAPS** | | | | |
| Shelter | 3 (42.9) | 4 (57.1) | 0.236 | Reference |
| Outpatient | 21 (75.0) | 7 (25.0) | | 0.25 (0.04–1.39) |
| Shelter and outpatient | 20 (62.5) | 12 (37.5) | | 0.45 (0.08–2.37) |
| **Mental disorder** | | | | |
| Anxiety | 6 (75.0) | 2 (25.0) | 0.552 | Reference |
| Mood | 9 (81.8) | 2 (18.2) | | 0.67 (0.06–6.84) |
| Related to substance use | 23 (62.2) | 14 (37.8) | | 1.83 (0.36–13.66) |
| Others | 6 (54.5) | 5 (45.5) | | 2.50 (0.37–22.74) |
| **ICD–10 categories for mental disorders** | | | | |

| | | | | |
|---|---|---|---|---|
| F00–F09 | 2 (66.7) | 1 (33.3) | 0.606 | - |
| F10–F19 | 23 (62.2) | 14 (37.8) | | - |
| F20–F29 | 3 (50.0) | 3 (50.0) | | - |
| F30–F39 | 9 (81.8) | 2 (18.2) | | - |
| F40–F48 | 6 (75.0) | 2 (25.0) | | - |
| F60–F69 | 1 (100.0) | 0 (0.0) | | - |
| F70–F79 | 0 (0.0) | 1 (100.0) | | - |
| **Co–infection** | | | | |
| No | 27 (69.2) | 12 (30.8) | 0.603 | Reference |
| Yes | 17 (60.7) | 11 (39.3) | | 1.46 (0.52–4.07) |
| **Adherence to ART** | | | | |
| No | 10 (100.0) | 0 (0.0) | 0.026 | - |
| Yes | 33 (64.7) | 18 (35.3) | | - |

Data source: Laboratory Test Control System of the National Network for CD4+/CD8+ Lymphocyte Counting and data obtained by reviewing outpatient and hospital charts of follow–up services.

*Fisher's exact test.

OR: Odds Ratio; 95% CI: 95% Confidence Interval; SD: Standard Deviation; IQR: Interquantile Range; CAPS: Psychosocial Care Centers; RAPS: Psychosocial Care Network; ICD–10: 10th edition of the International Classification of Diseases (ICD–10).

## Supplementary Table S2. CD4+ cells count according to clinical–epidemiological data

| | CD4+ cells count (cells/mm$^3$) | | | | *p*–value* | OR (95% CI) |
|---|---|---|---|---|---|---|
| | **Less than 200 (%)** | **201 to 350 (%)** | **350 to 500 (%)** | **Higher than 500 (%)** | | |
| **Age** | | | | | | |
| Mean (SD) | 42.5(8.6) | 40.8 (16.0) | 41.0 (11.0) | 39.4 (12.6) | 0.850 | 0.98 (0.93–1.04) |
| Median (IQR) | 42.0 (38.0–46.5) | 38.5 (29.5–42.8) | 40.5 (32.5–50.0) | 41.0 (28.0–48.0) | | |
| **Sex** | | | | | | |
| Female | 4 (20.0) | 3 (15.0) | 2 (10.0) | 11 (55.0) | 0.915 | Reference |
| Male | 7 (14.6) | 7 (14.6) | 8 (16.7) | 26 (54.2) | | 1.46 (0.35–5.56) |
| **Race or skin color** | | | | | | |
| White | 4 (16.0) | 5 (20.0) | 7 (28.0) | 9 (36.0) | 0.292 | - |
| Black | 0 (0.0) | 2 (33.3) | 0 (0.0) | 4 (66.7) | | - |
| Yellow | 1 (16.7) | 0 (0.0) | 0 (0.0) | 5 (83.3) | | - |
| Brown | 5 (17.9) | 3 (10.7) | 3 (10.7) | 17 (60.7) | | - |
| **Educational level** | | | | | | |
| Elementary school | 1 (4.8) | 2 (9.5) | 5 (23.8) | 13 (61.9) | 0.072 | - |
| High school | 2 (40.0) | 0 (0.0) | 1 (20.0) | 2 (40.0) | | - |
| Higher education | 4 (40.0) | 0 (0.0) | 1 (10.0) | 5 (50.0) | | - |
| Illiterate | 1 (50.0) | 1 (50.0) | 0 (0.0) | 0 (0.0) | | - |
| **Regular follow–up at CAPS** | | | | | | |
| No | 8 (19.5) | 8 (19.5) | 4 (9.8) | 21 (51.2) | 0.268 | Reference |
| Yes | 3 (11.1) | 2 (7.4) | 6 (22.2) | 16 (59.3) | | 1.94 (0.50–9.56) |
| **Homeless** | | | | | | |
| No | 9 (17.3) | 7 (13.5) | 8 (15.4) | 28 (53.8) | 0.945 | Reference |
| Yes | 2 (12.5) | 3 (18.8) | 2 (12.5) | 9 (56.2) | | 1.47 (0.33–10.36) |
| **Abuse of alcohol** | | | | | | |

| | | | | | | |
|---|---|---|---|---|---|---|
| No | 6 (17.1) | 3 (8.6) | 7 (20.0) | 19 (54.3) | 0.376 | Reference |
| Yes | 5 (15.2) | 7 (21.2) | 3 (9.1) | 18 (54.5) | | 1.16 (0.31–4.43) |
| **Use of drugs** | | | | | | |
| No | 7 (18.4) | 6 (15.8) | 6 (15.8) | 19 (50.0) | 0.870 | Reference |
| Yes | 4 (13.3) | 4 (13.3) | 4 (13.3) | 18 (60.0) | | 1.47 (0.40–6.12) |
| **Regular follow–up in the specialized outpatient infectious disease facility** | | | | | | |
| No | 9 (25.0) | 9 (25.0) | 5 (13.9) | 5 (13.9) | 0.009 | Reference |
| Yes | 2 (7.1) | 1 (3.6) | 5 (17.9) | 20 (71.4) | | 4.33 (1.00–30.23) |
| **Suicidal ideation** | | | | | | |
| No | 10 (22.2) | 7 (15.6) | 7 (15.6) | 21 (46.7) | 0.208 | Reference |
| Yes | 1 (4.3) | 3 (13.0) | 3 (13.0) | 16 (69.6) | | 6.29 (1.09–119.40) |
| **Service regimen at RAPS** | | | | | | |
| Shelter | 1 (14.3) | 2 (28.6) | 0 (0.0) | 4 (57.1) | 0.692 | Reference |
| Outpatient | 4 (13.8) | 5 (17.2) | 6 (20.7) | 14 (48.3) | | 1.04 (0.05–8.85) |
| Shelter and outpatient | 6 (18.8) | 3 (9.4) | 4 (12.5) | 19 (59.4) | | 0.72 (0.03–5.46) |
| **Mental disorder** | | | | | | |
| Anxiety | 0 (0.0) | 1 (12.5) | 2 (25.0) | 5 (62.5) | 0.0071 | - |
| Mood | 0 (0.0) | 2 (16.7) | 3 (25.0) | 7 (58.3) | | - |
| Related to substance use | 5 (13.5) | 6 (16.2) | 5 (13.5) | 21 (56.8) | | - |
| Others | 6 (54.5) | 1 (9.1) | 0 (0.0) | 4 (36.4) | | - |
| **ICD–10 categories for mental disorders** | | | | | | |
| F00–F09 | 2 (66.7) | 0 (0.0) | 0 (0.0) | 1 (33.3) | 0.236 | - |
| F10–F19 | 5 (13.5) | 6 (16.2) | 5 (13.5) | 21 (56.8) | | - |
| F20–F29 | 3 (50.0) | 1 (16.7) | 0 (0.0) | 2 (33.3) | | - |
| F30–F39 | 0 (0.0) | 2 (16.7) | 3 (25.0) | 7 (58.3) | | - |
| F40–F48 | 0 (0.0) | 1 (12.5) | 2 (25.0) | 5 (62.5) | | - |

| | | | | | | |
|---|---|---|---|---|---|---|
| F60–F69 | 0 (0.0) | 0 (0.0) | 0 (0.0) | 1 (100.0) | | - |
| F70–F79 | 1 (100.0) | 0 (0.0) | 0 (0.0) | 0 (0.0) | | - |
| **Co–infection** | | | | | | |
| No | 2 (5.1) | 6 (15.4) | 4 (10.3) | 27 (69.2) | 0.007 | Reference |
| Yes | 9 (31.0) | 4 (13.8) | 6 (20.7) | 10 (34.5) | | 0.12 (0.02–0.52) |
| **Adherence to ART** | | | | | | |
| No | 0 (0.0) | 0 (0.0) | 2 (20.0) | 8 (80.0) | 0.208 | - |
| Yes | 10 (19.6) | 8 (15.7) | 8 (15.7) | 25 (49.0) | | - |

Data source: Laboratory Test Control System of the National Network for CD4+/CD8+ Lymphocyte Counting and data obtained by reviewing outpatient and hospital charts of follow–up services.

*Fisher's exact test.

OR: Odds Ratio; 95% CI: 95% Confidence Interval; SD: Standard Deviation; IQR: Interquantile Range; CAPS: Psychosocial Care Centers; RAPS: Psychosocial Care Network; ICD–10: 10th edition of the International Classification of Diseases (ICD–10).
